# The single-nucleotide polymorphisms in *CHD5* affect the prognosis of patients with hepatocellular carcinoma

**DOI:** 10.18632/oncotarget.23812

**Published:** 2017-12-26

**Authors:** Xiao Zhu, Qingming Kong, Liwei Xie, Zhihong Chen, Hongmei Li, Zhu Zhu, Yongmei Huang, Feifei Lan, Haiqing Luo, Jingting Zhan, Hongrong Ding, Jinli Lei, Qin Xiao, Weiming Fu, Wenguo Fan, Jinfang Zhang, Hui Luo

**Affiliations:** ^1^ Guangdong Provincial Key Laboratory of Medical Molecular Diagnostics, Dongguan Scientific Research Center, Guangdong Medical University, Dongguan, China; ^2^ Immunity and Biochemical Research Lab, Zhejiang Academy of Medical Sciences, Hangzhou, China; ^3^ Guangdong Institute of Microbiology, Guangzhou, China; ^4^ Institute of Marine Medicine Research, Guangdong Medical University, Zhanjiang, China; ^5^ Forensic Identification Institute, Guangdong Women and Children Hospital, Guangzhou, China; ^6^ The Affiliated Hospital Cancer Center, Guangdong Medical University, Zhanjiang, China; ^7^ Department of Blood Transfusion, Peking University Shenzhen Hospital, Shenzhen, China; ^8^ School of Pharmaceutical Sciences, Southern Medical University, Guangzhou, Guangdong, China; ^9^ Guanghua School of Stomatology, Hospital of Stomatology, Sun Yat-sen University, Guangzhou, China; ^10^ Cancer Center, Medical College of Georgia, Georgia Health Sciences University, Augusta, GA, USA; ^11^ Institute of Bioinformatics, University of Georgia, Athens, GA, USA; ^12^ Key Laboratory of Orthopaedics and Traumatology, The First Affiliated Hospital of Guangzhou University of Chinese Medicine, The First Clinical Medical College, Guangzhou University of Chinese Medicine, Guangzhou, China

**Keywords:** hepatocellular carcinoma, chromodomain-helicase-DNA-binding protein 5, prognosis, single-nucleotide polymorphisms, linkage disequilibrium

## Abstract

Previous studies showed that the low expressions of chromodomain-helicase-DNA-binding protein 5 (CHD5) were intensively associated with deteriorative biologic and clinical characteristics as well as outcomes in many tumors. The aim of this study is to determine whether *CHD5* single nucleotide polymorphisms (SNPs) contribute to the prognosis of hepatocellular carcima (HCC). The SNPs were selected according to their linkage disequilibrium (LD) in the targeted next-generation sequencing (NGS) and then genotyped with TaqMan probers. We revealed a rare haplotype AG in *CHD5* (SNPs: rs12564469-rs9434711) was markedly associated with HCC prognosis. The univariate and multivariate regression analyses revealed the patients with worse overall survival time were those with tumor metastasis and haplotype AG, as well as cirrhosis, poor differentiation and IV-TNM stage. Based on the available public databases, we discovered the significant association between haplotype AG and *CHD5* mRNA expressions only existed in Chinese. These data proposed that the potentially genetic haplotype might functionally contribute to HCC prognosis and *CHD5* mRNA expressions.

## INTRODUCTION

Liver cancer is a primary malignancy of the hepar and caused by chronic liver disease and cirrhosis due to hepatitis B, hepatitis C, aflatoxin, alcohol or non-alcoholic fatty liver disease, et al.. The most common types are hepatocellular carcima (HCC), which makes up 80% of all cases, and cholangiocarcinoma [1].

*Chromodomain-helicase-DNA-binding protein 5* (*CHD5*) gene encodes an enzyme that in humans named CHD5 protein. CHD5 is a member of the chromatin organizing modulator domain superfamily, contains two zinc-binding plant homeodomain (PHD) fingers, chromo motifs, and a helicase domain [2]. It has two N-terminal chromodomains and a less well defined C-terminal DNA binding domain, which is approximately 1000 amino acids larger than sequences from members of subfamily I and II [3].

Previous studies suggest that genetic and epigenetic alterations are both involved in inhibiting cell proliferation, migration and invasion, as well as induce apoptosis in some cancer development, such as renal cell carcinoma [4], neuroblastomas [5]. Reduced *CHD5* expression is associated with unfavorable clinical features and outcome of cancer patients [6]. Resent studies also showed that *CHD5* acted as a tumor suppressor in HCC [7]. Identification of alterations could be helpful to unravel the mechanisms underlying carcinogenesis and develop potential biomarkers for cancer screening and prognosis prediction. This work will study the association of its single-nucleotide polymorphisms (SNPs) and HCC patients’ prognosis was not studied yet.

## RESULTS

### Univariate and multivariate regression models of prognostic factors

**Figure 1:**
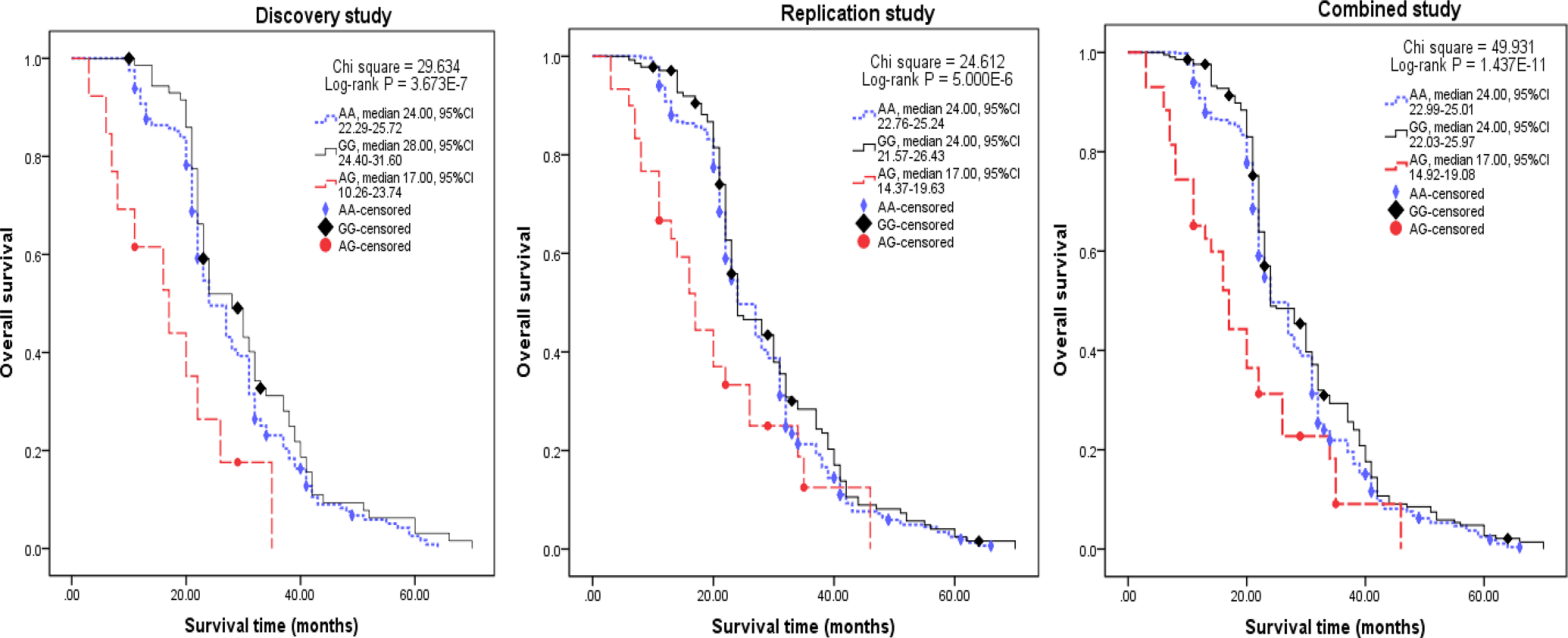
Kaplan-Meier survival curves according to haplotypes (block 3) in the discovery, replication and combined studies *P* value was calculated using a log-rank test.

Five-year overall survivals for patients were 7.50% in the discovery study, 8.38% in the replication study, and 8.08% in the combined study. We investigated the association of patients’ overall survival. Patients with haplotype AG had a shorter survival time (median 17.00 months) in the discovery, replication and combined studies, with conspicuous log-rank *P* values (3.673 × 10^-7^, cases in the discovery study; 5.000 × 10^-6^, cases in the replication study; and 1.437 × 10^-11^, cases in the combined study) (Figure 1). We analyzed the associations of overall survival and the clinical variables, and found age, cirrhosis, differentiation, metastasis and TNM stages were associated with HCC survival in univariate analysis (Table 1).

**Table 1:** Clinical and laboratory features of the subjects and univariate analysis for overall survival

Variables	Discovery study			Replication study			Combined study		
	*n*	5-years survival rates %	Log-rank *P*	*n*	5-years survival rates %	Log-rank *P*	*n*	5-years survival rates %	Log-rank *P*
Age (years)									
< 55	98	5.10		213	4.23		311	4.50	
≥ 55	182	8.79	0.087	336	11.01	0.036	518	10.23	0.013
Gender									
Females	53	9.43		125	9.60		178	9.55	
Males	227	7.05	0.405	424	8.02	0.747	651	7.68	0.584
Smoking									
No	176	7.95		296	10.47		472	9.53	
yes	99	7.07	0.942	231	6.49	0.183	330	6.67	0.297
Drinking									
No	177	8.47		311	11.25		488	10.25	
Yes	95	6.32	0.401	210	5.24	0.059	305	5.57	0.131
HBV									
HBsAg (-)	56	10.71		130	12.30		186	11.83	
HBsAg(+)	224	6.70	0.141	419	7.16	0.131	643	7.00	0.136
Serum AFP									
< 25 ng/ml	47	6.38		118	10.17		165	9.09	
≥ 25 ng/ml	233	7.73	0.869	431	7.89	0.495	664	7.83	0.623
Tumor size (cm)									
≤ 5	65	10.77		139	11.51		204	11.27	
> 5, ≤ 10	93	8.60	0.538	273	7.33	0.169	366	7.65	0.215
>10	122	4.92	0.037	137	7.30	0.104	259	6.18	0.058
Cirrhosis									
No	16	25.00		38	15.79		54	18.52	
Yes	260	6.54	0.005	504	7.94	0.027	764	7.46	< 0.001
Tumor morphology									
No residual tumor	19	15.79		43	13.95		62	14.52	
Uninodular tumor	55	9.09	0.192	89	8.99	0.744	144	9.03	0.376
Multinodular	107	7.48	0.111	228	7.02	0.078	335	7.16	0.090
tumor									
Massive tumor	92	5.43	0.035	168	9.52	0.301	260	8.08	0.124
Differentiation									
Well	31	48.39		77	25.97		108	32.41	
Moderate	78	3.85	< 0.001	195	7.69	< 0.001	273	6.59	< 0.001
Poor	171	1.75	< 0.001	277	3.97	< 0.001	448	3.13	< 0.001
Metastasis									
Abscent	81	22.22		189	19.58		270	20.37	
Present	193	1.55	< 0.001	347	2.59	< 0.001	540	2.22	< 0.001
TNM stage									
I	53	16.98		148	14.86		201	15.42	
II	95	7.37	0.039	230	6.52	0.051	325	6.77	0.025
III	64	4.69	0.006	110	5.45	0.025	174	5.17	0.004
IV	68	2.94	< 0.001	61	4.92	0.001	129	3.88	< 0.001

AFP, alpha fetoprotein; TNM, tumor, node, metastasis-classification.

For further validating the importance of the regression variables, a re-estimation of multivariate model analysis was carried out for the above 8 characteristics in the discovery, replication and/or combined studies. We found only the percentage of age made a reverse significant but marginal contribution (≥ 55 *vs.* < 55: OR = 0.87 < 1, *P* = 0.047). We found tumor metastasis, haplotype AG, cirrhosis, poor differentiation and IV-TNM stage were the prognostic factors of HCC (OR > 1, *P* < 0.05; Table 2).

**Table 2:** Cox multivariate regression of potential prognostic factors for overall survival

Variables	Discovery study		Replication study		Combined study	
	HR (95% CI)	*P*	HR (95%CI)	*P*	HR (95%CI)	*P*
Age (years) ≥ 55 vs. < 55	/	/	0.95 (0.86–1.07)	0.197	0.87 (0.77–0.98)	0.047
Tumor size > 10 cm vs. ≤ 5 cm	1.02 (0.83–1.23)	0.563	/	/	/	/
Cirrhosis Yes vs. No	1.32 (1.16–1.49)	0.016	1.10 (1.01–1.28)	0.049	1.82 (1.19–2.72)	0.008
Tumor morphology						
Massive tumor vs. No residual tumor	1.06 (0.86–1.2 8)	0.404	/	/	/	/
Differentiation						
Moderate vs. Well	1.03 (0.75–1.44)	0.585	1.74 (0.96–1.25)	0.133	1.34 (0.83–1.60)	0.127
Poor vs. Moderate	6.09 (3.71–8.85)	8.062 × 10^-4^	2.57 (1.37–3.92)	0.009	5.94 (2.11–9.52)	0.002
Metastasis Present vs. Abscent	8.52 (3.19–16.37)	1.990 × 10^-4^	5.76 (2.54–10.71)	9.175 × 10^-4^	14.08 (2.88–31.38)	2.636 × 10^-5^
TNM stage						
II vs. I	1.09 (0.90–1.34)	0.179	/	/	1.15 (0.96–1.28)	0.064
III vs. I	1.17 (0.97–1.41)	0.074	1.12 (0.92–1.29)	0.124	1.45 (0.95–1.80)	0.093
IV vs. I	3.35 (1.23–5.69)	0.006	2.95 (1.25–4.73)	0.017	3.72 (1.45–5.95)	0.009
Haplotype (block 3)						
AG vs. AA+GG	6.58 (3.56–9.55)	7.827 × 10^-4^	5.21 (2.34–8.17)	0.003	11.86 (3.37–21.14)	8.569 × 10^-5^

### Evolutional conserved regions (ECRs), mutations, expressions of CHD5 in silico

**Figure 2:**
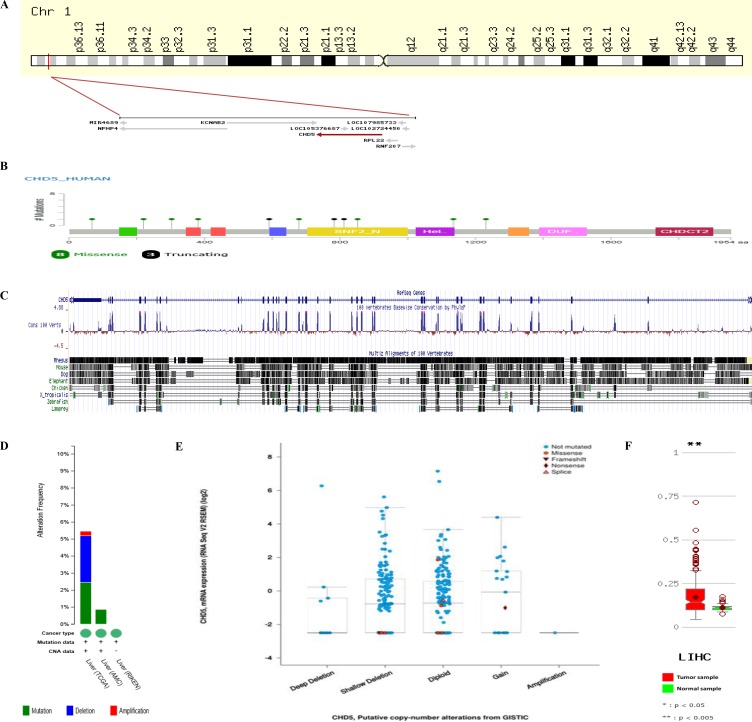
CHD5 structure, mutations and expressions in silico (**A**) *CHD5* in chromosome 1p36.31 and its transcriptional direction from the *National Center for Biotechnology Information* (NCBI, https://www.ncbi.nlm.nih.gov/). (**B**) *CHD5* gene mutations from TCGA (https://cancergenome.nih.gov/). The Cancer Genome Atlas (TCGA) hepatocellular carcinoma datasets and the related clinicopathologic information of the included patients were obtained from the cBioPortal (http://www.cbioportal.org/) for Cancer Genomics generated by Memorial Sloan-Kettering Cancer Center. (**C**) *CHD5* gene ECR generated from the UCSC Genome Browser (https://genome.ucsc.edu/). (**D**) the deletions of CHD5 from TCGA *in silico* analysis. (**E**) copy-number alterations from GISTIC (https://software.broadinstitute.org/software/cprg/?q = node/31). By separating somatic copy-number alterations (SCNAs) profiles into underlying arm-level and focal alterations, GISTIC estimates the background rates for each category as well as defines the boundaries of SCNA regions. (**F**) *CHD5* expressions in HCC samples and matched normal samples comparing of average beta value from TCGA.

On the basis of the information in UCSC database and NCBI, the *Homo sapiens CHD5* gene is located on chromosome 1p36.31 (Figure 2A), and its transcript is composed of 42 exons (which encodes a protein of 1954 amino acids) (Figure 2B). We identified an ECR by local and global alignment programs, which indicated that *CHD5* is evolutionarily conserved (especially in the exons) among diverse species (Figure 2C).

When looking at the incidence of mutations in the *CHD5* encoding the protein in tumor samples, using TCGA and the web tool cBioPortal for visualization and analysis, we identified a total of 11 mutations in *CHD5* mRNA from TCGA dataset, consisting of 8 missense and 3 truncating mutations. Eight tumor samples (H112501, H072969, TCGA-BC-A10W-01, TCGA-G3-AAV0-01, TCGA-DD-AADF-01, TCGA-DD-AACQ-01, TCGA-GJ-A9DB-01 and TCGA-G3-A25Z-01) had a different missense mutation, one sample (TCGA-4R-AA8I-01) had a splice mutation (X812_splice), one sample (TCGA-DD-A1EG-01) had a deletion (D783Tfs*7), and one sample (TCGA-CC-A7IK-01) had a nonsense mutation (W591*). Of them, three of the mutations in the CHD5 gene are localized to the amino-median of the SNF2_N domain (Figure 2B). And the deletions were only found in the data from the National Cancer Institute in HCC samples, which did not found in the East Asian patients (Figure 2D). In Figure 2E, copy-number alterations from GISTIC [8] showed those who have shallow deletion and diploid have higher CHD5 expressions in the Western population. Compared with the normal samples (*paracarcinoma tissues),* HCC tissues had a higher expression (*P* < 0.005; Figure 2F).

### Correlating block 3 haplotypes and phenotype to predict HCC prognosis

**Figure 3:**
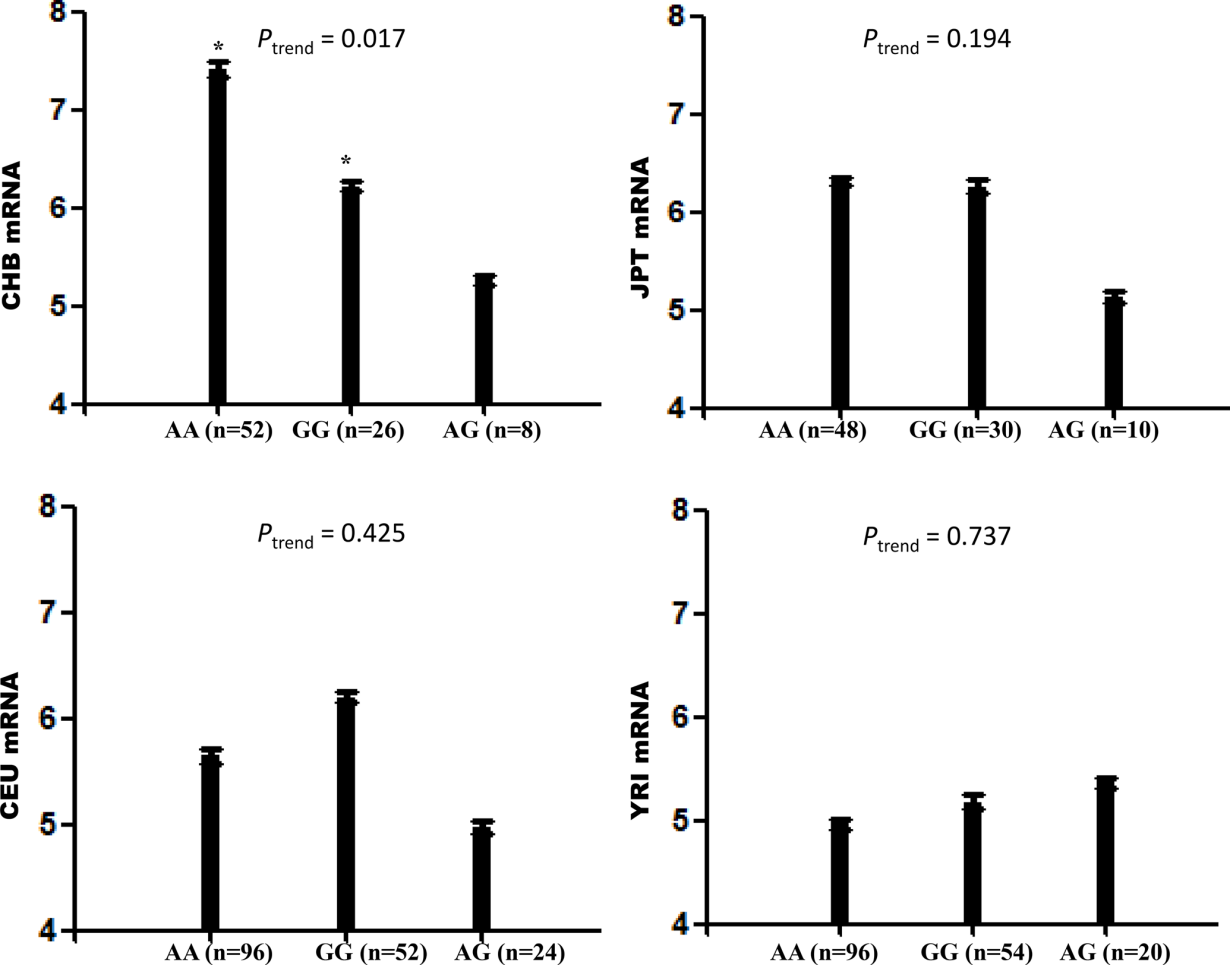
Correlation between CHD5 mRNA expressions and haplotypes (block 3) from EBV-transformed B lymphoblastoid cell lines from different populations available by SNPexp

According to the next generation RNA-seq from TCGA, we found that CHD5 expressed in almost all human normal tissues, cells and fluids (Supplementary Figure 1). In the haplotype-phenotype correlation analysis, we found statistically significant trends for the haplotypes effect on *CHD5* mRNA expression in CHB (45 unrelated Han Chinese in Beijing, *P*_AG/AA_ = 0.041, *P*_AG/GG_ = 0.027, *P*_trend_ = 0.017). But the same trend was not found in JPT (45 unrelated Japanese in Tokyo, *P*_trend_ = 0.194), CEU (90 Utah residents from northern and western Europe, *P*_trend_ = 0.425), and YRI (90 Yoruba in Ibadan, Nigeria, *P*_trend_ = 0.737; Figure 3). This also suggested that CHD5 block 3 might be a causative loci and therapeutic target for HCC in Chinese.

## DISCUSSION

The progression of HCC from diagnosis to death is double-quick, with patients dying within several months after diagnosis. This disease is almost asymptomatic in the early stages and the patients are unaware of the disease until the later stages, which the tumor has already metastasized. Most of the patients cannot undergo surgery because the tumors are too advanced for resection when diagnosed. Also, the recurrence rate of this tumor is very high after surgery [9].

With the recent advances in molecular biology, important discoveries were found. As a chromatin-remodeling protein, CHD5 could bind DNA through histones and regulate gene transcription. It could positively regulate H3K27me3 thereby precisely inhibiting genes that promote the cell proliferation and differentiation in nerve cells or non-neuronal cells [10]. Furthermore, the downstream genes activated may include CDKN2A that positively regulates the p53/TP53 pathway, which in turn, prevents cell proliferation [11, 12].

Previous studies revealed that truncation mutations of *CHD5* occur in neuroblastomas [13]. For this reason, we predicted that mutations in the PHD might lead to the loss or gain of function of CHD5 proteins, thereby influencing patients’ overall survival and prognosis. Therefore, we studied the overall survival in two cases and combined them.

We found a significant trend for the effect of haplotype AG on *CHD5* transcript expressions in *Han* Chinese but not other populations, indicating that this haplotype may be a potential genetic determinant and therapeutic target for HCC in Chinese. Haplotype AG (block 3) was strikingly associated with a poor prognosis in HCC patients. Forward progressive selection-univariate analysis, using the training set, resulted in the options of the coefficients, all with *P* values < 0.05. In the present study, we found that the prognosis of patients metastatic diseases with haplotype AG is no better than that of patients without metastasis and with other haplotypes. Haplotypes are a set of alleles of a group of closed linked genetic markers, which are usually inherited as a unit [14]. An individual inherited a complete haplotype from each parent, therefore could narrow down the numbers of investigated markers.

Further genotype-phenotype analyses showed that the lower level of *CHD5* mRNA expression was associated with the haplotype AG in Chinese population but not in Japanese, Caucasian and African populations. Given that lower CHD5 mRNA expression levels were correlated to haplotype AG in some target tissues, joined the above survival analyses, it is reasonable to surmise that lower *CHD5* mRNA expression levels may be associated with shorter survival time in Chinese populations. However, the underlying mechanisms are still unintelligible. Recent studies have showed that *CHD5* expression is silenced by epigenetical hypermethylation in the gene promoter in some tumors including HCC and gastric cancer [7, 15, 16]. The *CHD5* hypermethylation may implicate chromatin dynamics and cancer-associated pathways. The hypothesis that *CHD5* works as a tumor-suppressing gene in HCC might simplify the tanglesome landscape of a chromatin remodeler. Many of these enzymes, such as CHD4 and BRG1, including their mutations and SNPs, display ambiguous roles during oncogenesis. As a chromatin remodeling protein, *CHD5* could regulate developmentally ATP-dependent chromatin remodeling [17]. The function change of protein may influence on patients survival time.

We do not yet conclude the mechanisms of how it is impacting on the expression of CHD5 protein in HCC tissues. Taken these limitations into consideration, we recognized that further investigations in HCC patients needed to be implemented to find out the precise mechanisms.

## MATERIALS AND METHODS

### Ethics statement

The study was approved by the local ethics committee (Guangdong Medical University). All the relatives of patients provided written informed consent. The study was conducted according to the principles expressed in the Declaration of Helsinki.

### Study subjects

Firstly, 280 unrelated HCC patients (who were from Zibo Central Hospital in North China between 2006 and 2010) were recruited in the discovery study. Then, 549 HCC patients (who were from Peking University Shenzhen Hospital between 2007 and 2010, the First Affiliated Hospital, Sun Yat-Sen University between 2007 and 2015, and Cancer Hospital of Guangzhou Medical University between 2009 and 2011 in South China) were included in the replication study. The mean age (years) of the patients was all around 56 in two groups. Age distribution was throughout 30 to 75 years old. Furthermore, the ratio of male individuals is about 3.65 times higher than that of females in cases (combined study). All cases were *Han* origin Chinese and lived in China. The main features of the subjects included are summarized in Table 1.

At recruitment, each study participant (or his/her relative) was interviewed via a structured questionnaire, to obtain information on demographic characteristics, habits of alcohol drinking and cigarette smoking, as well as personal and family history of major chronic illnesses. Pack-years were calculated as the average number of pack of cigarettes consumed per day multiplied by the number of years a person has smoked [18]. We defined “former smokers” as the above who quitted smoking ≥ 1 year previously, and “current smokers” as the above who currently smoked or quitted smoking < 1 year ahead of the interview. We defined “never smokers” as those who had never smoked or had smoked for < 1 year. Ever drinkers who had stopped drinking more than one year previously were regarded as “former drinkers” and the others as “current drinkers”. The amount of each type of beverage (liquor/spirits, wine or beer) consumed during the year before the registration was reported. Daily ethanol consumption in grams was calculated rooted in the ethanol content of the beverage. One drink was regarded as 30 g of spirits (12.9 g of ethanol), 103 g of wine (12.3 g of ethanol), or 360 g of beer (12.6 g of ethanol) [19, 20]. The number of subjects in cases who drunk and/or used alcohol was much more than that in controls. Several studies on the tobacco or alcohol consuming in HCC patients have been conducted with conflicting results. In our research, the proportion of smoking or drinking has about 39.81% or 36.79% in HCC patients (combined study). However, if we want to know the effects of smoking or drinking in HCC, further studies in larger samples should be managed.

The serum laboratory tests, tumor characteristics, staging and prior therapy were collected according to the records of case history. The diagnostic criteria of HCC have been described as our previous study [21]. Briefly, the diagnosis of HCC was verified by either positive liver histologic findings, or rooted in the findings of medical imaging features indicative of HCC in at least two image examinations including abdominal ultrosound, high-resolution contrast enhanced computed tomography (CT), magnetic resonance imaging (MRI) and liver angiography, or by a single positive imaging technique added to serum α-fetoprotein level ≥ 25 μg/L. Those negative results in abdominal iconography or α-fetoprotein levels will be diagnostic by transparietal biopsy proof. The tumor, nodes, metastasis-classification (TNM) system was described as before [22].

### Patients follow up

Survival Data were gathered from the inpatient and outpatient records in the hospitals, primary physician’s offices, and/or patient or family contact. The duration of survival was from the date of carcinomatous diagnosis to the date of death, or last known date alive. The patients were followed up for a median time of five years. The log-rank test was used to judge the relationship between the haplotypes (block 3) and the prognosis of patients from the date of diagnosis to the end of follow-up. The Cox regression model was used to analyzed survival-time (time-to-event) outcomes on one or more predictors.

### Targeted sequencing, SNPs selection and genotyping

We sequenced whole CHD5 gene with next generation sequencing technology (Illumina Genome Analyzer) in 280 HCC samples and found total 164 SNPs in this gene including its 5’- and 3’-ends (Supplementary Figure 2). We defined haplotype blocks according to linkage disequilibrium (LD) using the Haploview program [23]. The SNPs selected following the haplotype blocks were rs12037962, rs11587, rs41307753 and rs3810989 (in block 1), rs2273041, rs2273040, rs2273038 and rs55930553 (in block 2), and rs12564469 and rs9434711 (in block 3) (Supplementary Figure 2). Then, genomic DNAs from all the other subjects (549 cases) were genotyped by TaqMan probes in Applied Biosystems ABI 7500 Fast System (Forster City, CA) for the selected two SNPs in haplotypic block 3 (rs12564469 and rs9434711).

### Comparative genomic analysis of CHD5 gene

To identify evolutionarily conserved regions (ECRs), we compared the Homo sapiens *CHD5* sequence (UCSC Genome Browser on Human Dec. 2013 (GRCh38/hg38) Assembly) with 44 vertebrate homologous sequences. The BLASTZ algorithm [24] and PhastCons [25] incorporated in the UCSC browser [26].

### Data mining of CHD5 mutations/deletions and mRNA expressions in HCCs

To portray the regulation model of CHD5 gene, the CHD5 mRNA expression data from The Cancer Genome Atlas (TCGA) hepatocellular carcinoma datasets and the related clinicopathologic information of the included patients were obtained from the cBioPortal for Cancer Genomics generated by Memorial Sloan-Kettering Cancer Center. For mRNA expression data, the relative expression of an individual gene and the distribution of a gene’s expression in a reference population were analyzed. Analysis of 442 HCC samples (TCGA, provisional), 231 HCC samples (from Asian Medical Center, Korea) [27], and 27 HCC samples (from RIKEN with whole-genome sequencing) [28] were performed *in silico*.

### Haplotype-phenotype correlation analysis

CHD5 expressions data in normal human tissues, cells and fluids were analyzed from the next-generation sequencing (GREx/Illumina Human BodyMap from TCGA). To further understand how the *CHD5* genetic variation influences its expression in tumor progress, we tried to examine the data from the HapMap Project consisting of 3.96 million SNP genotypes from 270 individuals of four ethnic groups (CHB, JPT, CEU, and YRI) and CHD5 mRNA expression levels from EBV-transformed B lymphoblastoid cell lines from the same 270 individuals available by SNPexp [29]. Finally, least squares analysis of variance (ANOVA) was performed to calculate statistical levels of CHD5 transcripts expression according to different haplotypes.

### Statistical analysis

All statistical tests were two-sided and *P* values less than 0.05 were considered statistically significant. SPSS 22.0 (SPSS, Chicago, IL) was used in this study. Kaplan–Meier survival curves and the log-rank test for trend were used to evaluate the relationship between the potential haplotypes and the HCC prognosis. Hazard ratios (HR) and 95% confidence intervals (CIs) for each factor in multivariate analysis were calculated from the Cox regression model.

## SUPPLEMENTARY MATERIALS FIGURES


