# Supplementary material for: The single-nucleotide polymorphisms in CHD5 affect the prognosis of patients with hepatocellular carcinoma
Source: Oncotarget. 2017 Dec 26;9(17):13222–30. doi: 10.18632/oncotarget.23812 (PMC5862573; doi:10.18632/oncotarget.23812)
Supplement: Supplementary file 1 [file oncotarget-09-13222-s001.pdf]

The single-nucleotide polymorphisms in *CHD5* affect the prognosis of patients with hepatocellular carcinoma

SUPPLEMENTARY MATERIALS

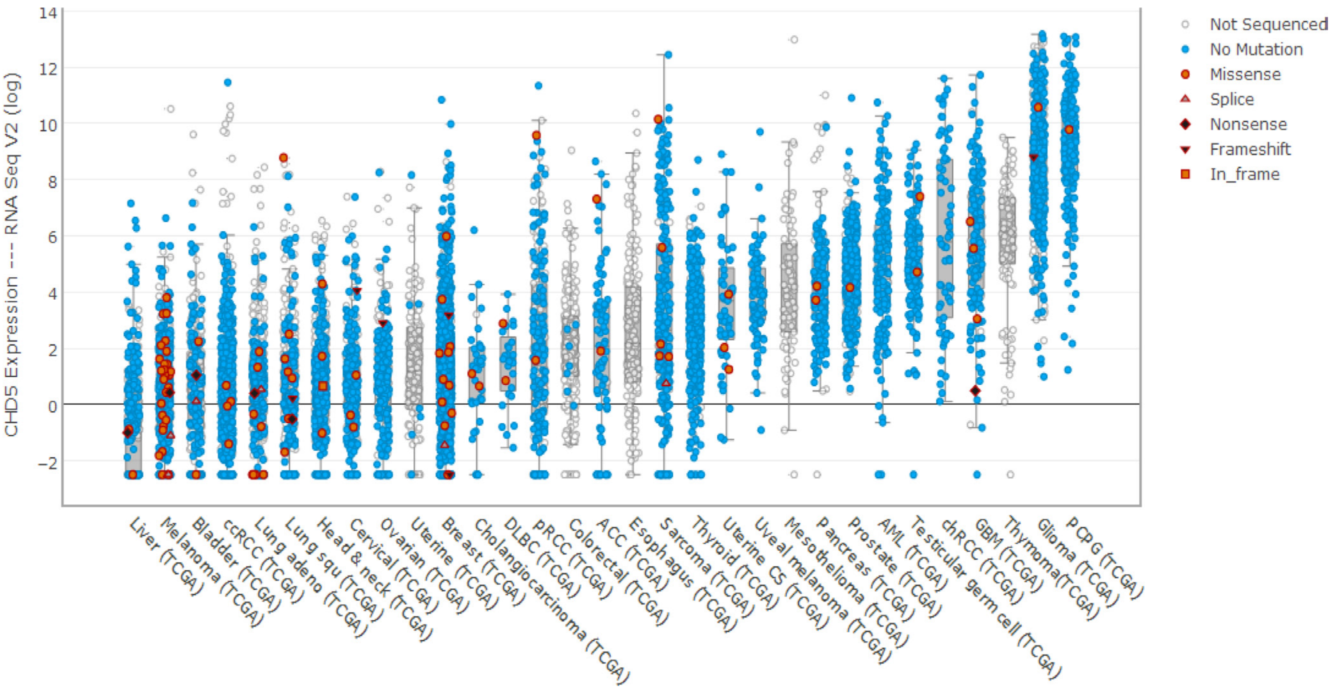

Supplementary Figure 1: CHD5 expressions in different human normal tissues, cells and fluids from TCGA.

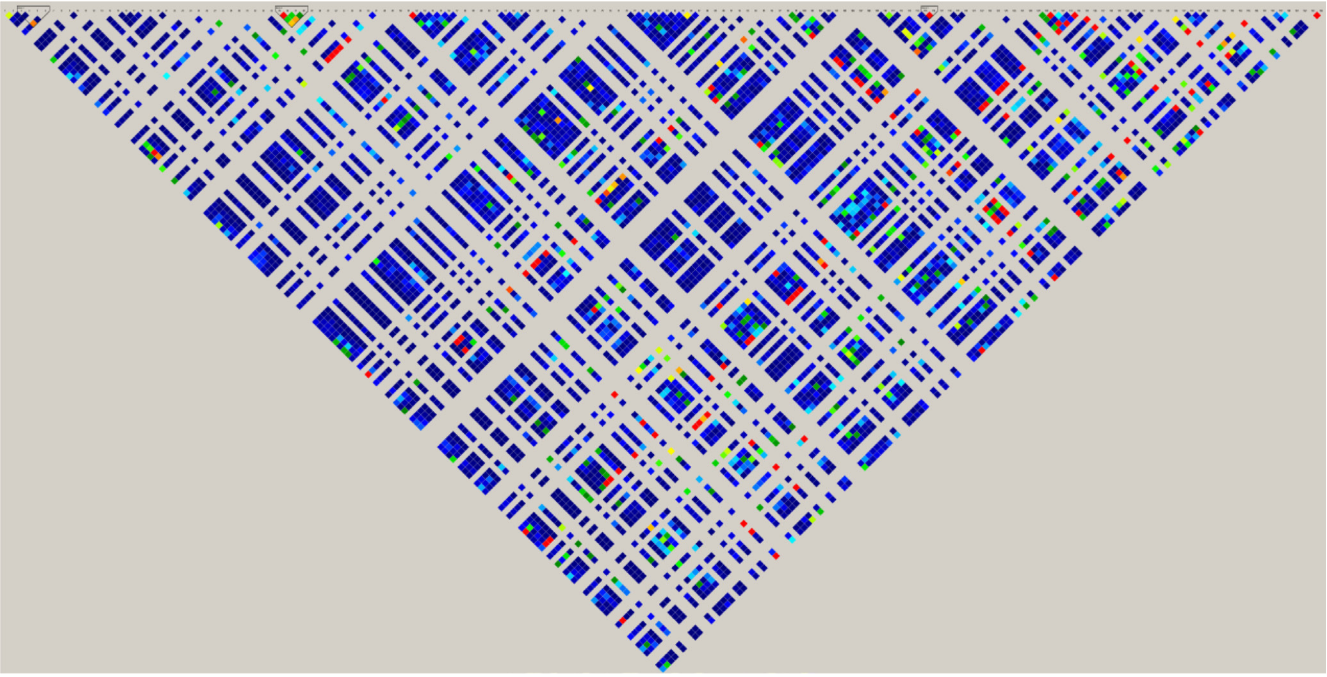

Supplementary Figure 2: Linkage disequilibrium of 164 SNPs from targeted next generation sequencing in CHD5 gene.
